# Structural MRI correlates of PASAT performance in multiple sclerosis

**DOI:** 10.1186/s12883-018-1223-0

**Published:** 2018-12-20

**Authors:** Jordi A. Matias-Guiu, Ana Cortés-Martínez, Paloma Montero, Vanesa Pytel, Teresa Moreno-Ramos, Manuela Jorquera, Miguel Yus, Juan Arrazola, Jorge Matías-Guiu

**Affiliations:** 1 Department of Neurology, San Carlos Health Research Institute (IdISSC), Universidad Complutense de Madrid, C/ Profesor Martín Lagos s/n, 28040 Madrid, Spain; 2 Department of Radiology, IdISSC, Universidad Complutense de Madrid, Madrid, Spain

**Keywords:** Cognitive impairment, Multiple sclerosis, PASAT, Voxel-based lesion symptom mapping, Voxel-based morphometry

## Abstract

**Background:**

The Paced Auditory Serial Addition Test (PASAT) is a useful cognitive test in patients with multiple sclerosis (MS), assessing sustained attention and information processing speed. However, the neural underpinnings of performance in the test are controversial. We aimed to study the neural basis of PASAT performance by using structural magnetic resonance imaging (MRI) in a series of 242 patients with MS.

**Methods:**

PASAT (3-s) was administered together with a comprehensive neuropsychological battery. Global brain volumes and total T2-weighted lesion volumes were estimated. Voxel-based morphometry and lesion symptom mapping analyses were performed.

**Results:**

Mean PASAT score was 42.98 ± 10.44; results indicated impairment in 75 cases (31.0%). PASAT score was correlated with several clusters involving the following regions: bilateral precuneus and posterior cingulate, bilateral caudate and putamen, and bilateral cerebellum. Voxel-based lesion symptom mapping showed no significant clusters. Region of interest–based analysis restricted to white matter regions revealed a correlation with the left cingulum, corpus callosum, bilateral corticospinal tracts, and right arcuate fasciculus. Correlations between PASAT scores and global volumes were weak.

**Conclusion:**

PASAT score was associated with regional volumes of the posterior cingulate/precuneus and several subcortical structures, specifically the caudate, putamen, and cerebellum. This emphasises the role of both cortical and subcortical structures in cognitive functioning and information processing speed in patients with MS.

**Electronic supplementary material:**

The online version of this article (10.1186/s12883-018-1223-0) contains supplementary material, which is available to authorized users.

## Background

The Paced Auditory Serial Addition Test (PASAT) is a useful cognitive tool with high sensitivity to sustained attention and information processing speed alterations [1]. It is one of the most frequently employed neuropsychological tests in patients with multiple sclerosis (MS), as it has been added to several widely used batteries in this setting, such as the Brief Repeatable Neuropsychological Battery (BRN-B), the Minimal Assessment of Cognitive Function in Multiple Sclerosis, and the Multiple Sclerosis Functional Composite scale [2–4].

In PASAT, patients have to add 60 pairs of digits by adding each digit to the immediately preceding one. Digits are usually presented every 3 s [1]. PASAT is considered to be a difficult and sometimes very stressful test, requiring a high level of concentration. However, it is highly sensitive to cognitive decline in patients with MS and has been found to be useful for evaluating information processing speed [5].

Although it is widely used for assessing MS, the neural basis of PASAT performance continues to be debated. Several previous articles have determined the correlation between PASAT performance and total brain volume and/or T2-weighted lesion volume [6]; but few studies have addressed the specific brain regions associated with the test. In this regard, Morgen et al. [7] correlated PASAT performance with atrophy of the prefrontal cortex, precentral gyrus, superior parietal cortex and right cerebellum in a study of 19 patients with MS and 19 controls. Sbardella et al. [8] correlated PASAT performance with the orbitofrontal cortex, and white matter tracts located in the corpus callosum, internal capsule, thalamic radiations, and cerebral peduncles. In contrast, Nocentini et al. [9] found no significant correlations between PASAT performance and brain regions in a cohort of 18 patients with MS. And very recently, Riccitelli et al. [10] found correlations between PASAT performance and atrophy of grey matter nuclei and several fronto-temporo-occipital regions in a large cohort of 177 patients with MS.

Neuropsychological tests are standardised tools used to evaluate different cognitive functions, each of which has more or less specific neural underpinnings. Understanding the neural basis of a cognitive test may improve our interpretation of test results in clinical practice [11]. This is especially relevant in MS due to the multifocal nature of the disease, which constitutes a challenge in the interpretation of neuropsychological assessments; and in the particular case of PASAT, which probably involves several cognitive functions [5].

Our aim was to study the neural basis of PASAT performance in a large series of 242 patients with MS. We used structural magnetic resonance imaging (MRI) to estimate global brain volumes and performed a voxel-based morphometry and lesion symptom mapping analysis in order to identify the relationship between PASAT performance and global and regional brain atrophy and white matter lesions.

## Methods

### Study population and ethics

The study included patients meeting the revised McDonald criteria for MS [12]. We excluded patients with other causes of cognitive impairment besides MS, such as other neurological (e.g. stroke, brain tumour), medical (e.g. cancer, B_12_ vitamin deficiency), or psychiatric disorders (e.g. major depression, bipolar disorder, psychosis). Our hospital’s Ethics Committee approved the research protocol; written consent was obtained from all participants.

### Neuropsychological assessment

PASAT was administered according to the manual by a trained neuropsychologist. The stimulus was presented using an audiotape. Single digits were presented every 3 s. The total number of correct responses was recorded. Results were considered to represent impairment when the number of correct responses was > 1.5 standard deviations (SD) below the mean according to age- and education-adjusted normative data from our setting [13].

The patients were also examined using a comprehensive, co-normed battery assessing the main cognitive functions. This battery has been described elsewhere [14] and includes the following tests: forward and backward digit span, Corsi block-tapping test, Trail Making Test (TMT) parts A and B, Symbol Digit Modalities Test (written version) (SDMT), Boston Naming Test (BNT), Judgement of Line Orientation (JLO), Rey-Osterrieth Complex Figure (ROCF) (copy and recall at 3 and 30 min), Free and Cued Selective Reminding Test (FCSRT), verbal fluencies (animals and words beginning with “p”, “m”, and “r” in 1 min), Stroop Color Word Interference Test, and Tower of London-Drexel version (ToL) [15]. The Beck Depression Inventory and the Fatigue Severity Scale were also administered [16, 17].

### MRI acquisition, preprocessing, and analysis

MRI was acquired using a 1.5 T scanner (Signa HDxt, GE Healthcare, Milwaukee, USA) including these sequences: a) T1-weighted 3D fast spoiled gradient-echo inversion recovery (repetition time [TR] 12 ms, echo time [TE] 2.3 ms, inversion time [TI] 400 ms; slice thickness 1 mm in 78 cases (32.2%) and 3 mm in 164 patients (67.8%); b) T2-weighted fluid-attenuated inversion recovery (FLAIR) (TR 9102 ms, TE 121 ms, TI 2260 ms; slice thickness 3 mm); c) T2-weighted double-echo fast spin-echo (FSE) (TR 2620 ms, TE 15/90 ms); d) T1-weighted post-contrast FSE sequence (TR 640 ms, TE 11.8 ms) following injection of gadoteric acid.

Image preprocessing and analysis were conducted using Statistical Parametric Mapping 8 (SPM8) (The Wellcome Trust Centre for Neuroimaging, Institute of Neurology, University College of London, UK) and the associated VBM8 and Lesion Segmentation Tool (LST) toolboxes [18]. LST is designed specifically for MS and performs a semi-automatic segmentation of T2-hyperintense white matter lesions using 3D-T1 and FLAIR sequences via a lesion-growth algorithm, in addition to lesion filling on T1-weighted images. Subsequently, 3D-T1 images were segmented into grey matter, white matter, and cerebrospinal fluid compartments, then normalised to the standard space of the Montreal Neurological Institute using the DARTEL template. Finally, images were smoothed at 8 mm full-width at half maximum. Preprocessing was performed blind to neuropsychological assessment data. Two expert neuroradiologists (MJ and MY) assessed the images and JAM-G conducted the statistical image analysis.

We calculated partial correlations between PASAT raw score and normalised brain volumes (white matter and grey matter fractions) and lesion burden, controlling for age, sex, and years of education. A multiple regression analysis was performed to estimate which brain regions were correlated with PASAT performance (raw score), using a voxel-based morphometry procedure with SPM8. Age, years of schooling, sex, protocol of 3D-T1 weighted acquisition, and total intracranial volume were included in the statistical model as nuisance covariates. In an additional analysis, depression was also added as a covariate. A false-discovery rate of *P* < 0.05 was considered statistically significant at cluster level. A minimum cluster size k = 100 was also used to avoid the multiple comparisons problem.

Normalised lesion maps of T2-hyperintense lesions detected in FLAIR sequences were smoothed at 8 mm full width at half maximum and then used to perform voxel- and region of interest (ROI)-based lesion symptom mapping. Voxel-based or ROI-based lesion symptom mapping is a method to analyse the relationship between localization of brain damage and a behaviour, which has been successfully used in cognitive neuroscience to advance in the identification of critical regions or networks for specific brain functions [19]. The “NiiStat” MATLAB® toolbox (9 October 2016 version) was used for these analyses [20]. The CAT atlas was used for the definition of white-matter ROIs [21]. Age, sex, and years of formal education were included as nuisance covariates. A minimum overlap of 15 subjects was considered, and 10,000 permutations were calculated to correct for multiple comparisons, using a *P*-value of < 0.05 as threshold.

### Statistical analysis

Descriptive results are shown as frequencies (percentages), means ± SD, or medians (interquartile ranges), as appropriate. The chi-square and two-sample *t* tests were used for comparisons between 2 independent samples. Correlations between PASAT performance and other quantitative variables were calculated using Pearson’s coefficient. A *P*-value of < 0.05 was considered statistically significant.

Statistical analysis was performed using the IBM® SPSS statistics package, version 20.0.

## Results

### Demographic, cognitive, and MRI variables

The 242 patients in the sample comprised 164 women (67.8%) and 78 men (32.2%) with a mean age of 45.35 ± 8.97 and 16.14 ± 2.89 years of schooling. According to clinical form of MS, 195 (80.6%) had relapsing-remitting, 30 (12.4%) secondary progressive, and 17 (7.0%) primary progressive MS. Median Expanded Disability Status Scale (EDSS) score was 2.0 (1–3.5).

Mean PASAT score was 42.98 ± 10.44 (range 16–60); scores were > 1.5 SD below the mean in 75 (31.0%) cases. There were no significant differences between patients with and without impairment in PASAT performance in terms of age (45.09 ± 9.1 vs 45.47 ± 8.93, *t* = − 0.303, *P* = 0.762), EDSS score (2.48 ± 1.81 vs 2.35 ± 1.87, *t* = 0.501, *P* = 0.617), T2 lesion load (14.291 ± 16.992 vs 10.861 ± 12.440, *t* = 1.57, *P* = 0.118), and normalised grey matter volume (0.42 ± 0.03 vs 0.42 ± 0.02, *t* = 0.133, *P* = 0.894). Level of schooling was slightly higher in the group with PASAT scores > 1.5 SD below the mean (16.95 ± 2.25 vs 15.77 ± 3.08, *t* = 3.33, *P* = 0.001).

PASAT performance was significantly correlated with most of the other cognitive tests. However, the size of the correlation was at least moderate (*r* > 0.4) with only the following tests: TMT-B (*r* = − 0.464, *P* < 0.0001), SDMT (*r* = 0.416, *P* < 0.0001), Stroop part B (*r* = 0.464, *P* < 0.0001), Stroop part C (*r* = 0.490, *P* < 0.0001), semantic verbal fluency (*r* = 0.408, *P* < 0.0001), phonemic verbal fluency “p” and “m” words (*r* = 0.444, *P* < 0.0001; *r* = 0.406, *P* < 0.0001, respectively). Correlations with the other tests were as follows (all *P* < 0.0001): digit span forward (*r* = 0.246), digit span backward (*r* = 0.310), Corsi test forward (*r* = 0.362), Corsi test backward (*r* = 0.367), TMT-A (*r* = − 0.354), Boston Naming Test (*r* = 0.293), ROCF copy accuracy (*r* = 0.366), ROCF memory at 3 min (*r* = 0.296), ROCF memory at 30 min (*r* = 0.349), FCSRT free recall 1 (*r* = 0.250), FCSRT total recall (*r* = 0.238), FCSRT delayed free recall (*r* = 0.319), FCSRT delayed total recall (*r* = 0.273), Stroop part A (*r* = 0.384), Tower of London correct moves (*r* = 0.358), and Judgement Line Orientation (*r* = 0.349). Regarding depression and fatigue, correlation with Beck Depression Inventory and Fatigue Severity Scale was *r* = − 0.233 (*P* < 0.0001) and *r* = − 0.156 (*P* = 0.015), respectively.

### Correlation with MRI global measures

PASAT raw score correlated negatively with white matter lesion volume (*r* = − 0.186, *P* = 0.004), and positively with grey matter volume (*r* = 0.272, *P* < 0.0001), white matter volume (*r* = 0.244, *P* < 0.0001), and total intracranial volume (*r* = 0.250, *P* < 0.0001). However, it was not correlated with normalised grey matter volume (*r* = 0.026, *P* = 0.688) or normalised white matter volume (*r* = 0.118, *P* = 0.068).

### Voxel-based morphometry results: Multiple regression analysis

**Fig. 1:**
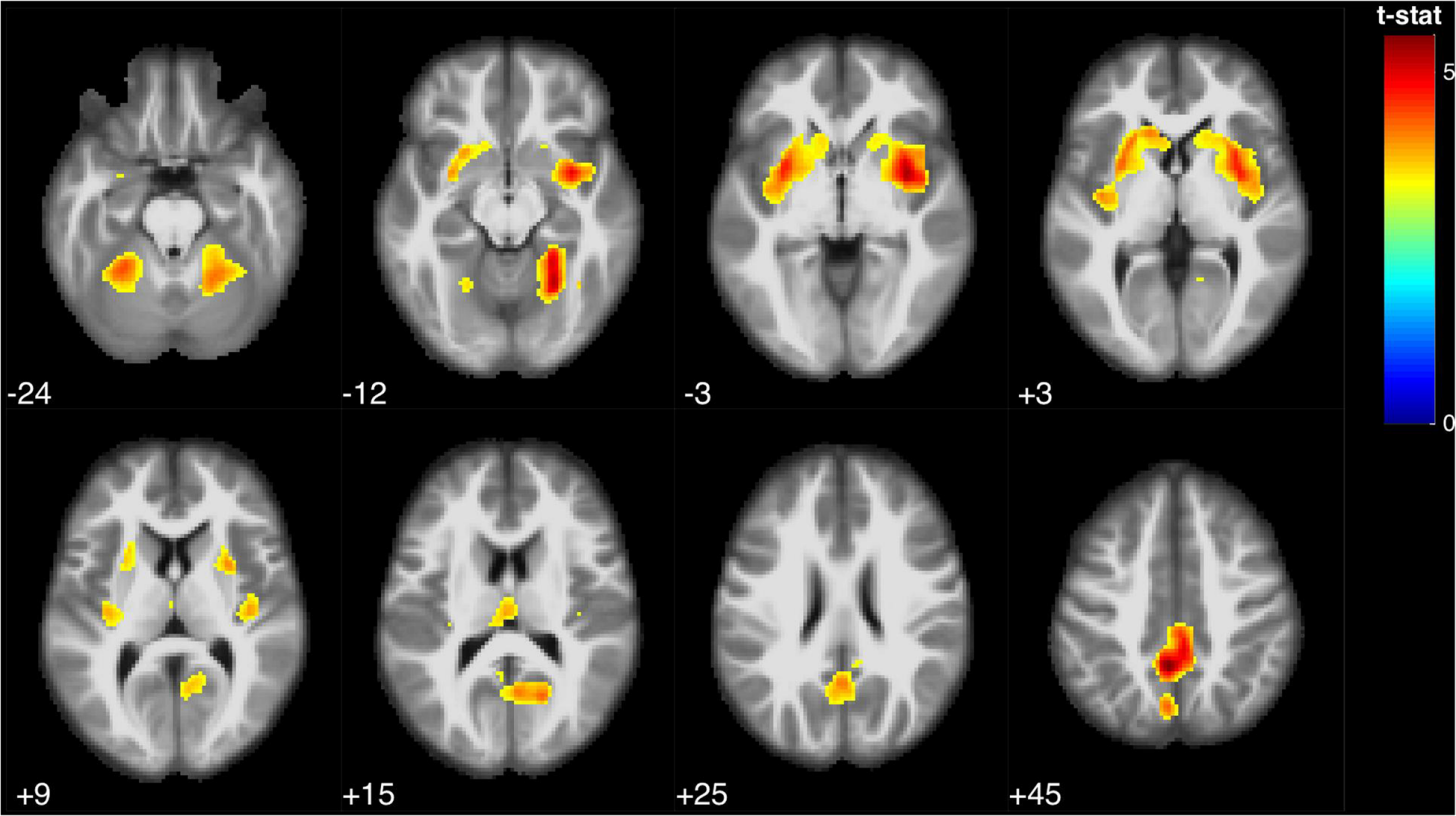
Statistical parametric map showing brain regions positively correlated with PASAT performance (FDR *p* < 0.05, k = 100), rendered on MRI template with neurological orientation

Voxel-based morphometry analysis showed that PASAT performance correlated with several clusters involving the following regions: bilateral precuneus and posterior cingulate, bilateral caudate and putamen, and bilateral anterior and posterior cerebellum (Table 1, Fig. 1). When controlling also depression scale as a covariate, results were very similar, showing an association of PASAT with several clusters involving precuneus/posterior cingulate, caudate/putamen, and cerebellum (Additional file 1).

**Table 1:** Voxel-based morphometry analysis. Multiple regression analysis showing correlations between PASAT and brain regions, using age, sex, years of education, and total intracranial volume as covariates. FDR corrected *p*-value < 0.05, k = 100

Brain region (Brodmann area)	MNI coordinates			T value	Z score	Cluster-level	Peak-level	K (number of voxels)
	x	y	z			*p*-value (FWE corrected)	*p*-value (FDR-corrected)	
Left and right precuneus and posterior cingulate [7, 31]	−4	− 48	45	5.53	5.36	< 0.0001	0.003	5556
	4	−39	43	5.04	4.91		0.003	
	18	−64	16	4.39	4.30		0.007	
Right insula [13], caudate and putamen	34	2	−3	5.30	5.15	< 0.0001	0.003	3972
	40	−18	9	3.99	3.92		0.0014	
	18	16	−12	3.34	3.30		0.0046	
Right cerebellum (anterior and posterior lobes)	24	−57	−12	5.19	5.04	< 0.0001	0.003	2719
	24	−46	−12	5.02	4.89		0.003	
	39	−58	−14	3.87	3.81		0.018	
Left insula [13], caudate and putamen	−32	3	−3	4.70	4.59	< 0.0001	0.004	3548
	−27	14	1	4.24	4.16		0.009	
	−22	21	3	4.19	4.11		0.010	
Left cerebellum (anterior lobe)	−27	−49	−24	4.43	4.34	0.013	0.006	1375
Left thalamus	−2	−19	15	3.91	3.84	0.532	0.017	324

### Voxel- and ROI-based lesion symptom mapping

Voxel-based lesion symptom mapping did not show any significant clusters. ROI-based analysis restricted to white matter regions showed five regions surviving the previously defined threshold: the left cingulum, corpus callosum, bilateral corticospinal tracts, and right arcuate fasciculus. When T2 total lesion volume was added to the statistical model as a regressor, no ROI reached statistical significance.

## Discussion

In this study, we used voxel-based morphometry and lesion symptom mapping methods to explore MRI correlates of PASAT performance in MS. Poorer performance was correlated with atrophy of several brain regions including the posterior cingulate and precuneus, caudate, putamen, and cerebellum. Previous studies analysing correlation with brain atrophy at the regional level have found conflicting results (see Table 2 for a summary of these studies) [22–24]. However, these studies generally included relatively small samples. In contrast, a recent large study by Riccitelli et al. [10] found PASAT performance to be correlated with atrophy of the bilateral thalamus, caudate and putamen, the right anterior cingulate, right superior frontal gyrus, and the right precentral, left superior temporal, and right fusiform gyri. Our study also found a correlation with the basal ganglia, as well as with the cerebellum and, interestingly, with the posterior cingulate and precuneus. The posterior cingulate/precuneus is a central node within the default mode network, and functional MRI analysis has demonstrated posterior cingulate and precuneus atrophy to be a good predictor of default mode network dysfunction in patients with MS [25]. Furthermore, some of the regions observed by Riccitelli et al. also belong to the default mode network; and this network has been associated with PASAT performance in fMRI studies [10, 26].

**Table 2:** Main studies evaluating the correlation between PASAT performance and MRI measures in multiple sclerosis

Author/year	Number of patients	MRI measures	Main results
Morgen et al., 2006 [7]	19 RRMS	T1	Correlation with bilateral prefrontal cortex, precentral gyrus, superior parietal cortex and right cerebellum
Dineen et al., 2009 [25]	37 MS	DTI (TBSS)	Correlation with fractional anisotropy in corpus callosum, parieto-occipital radiations of the forceps major, left cingulum, right inferior longitudinal fasciculus, left superior longitudinal fasciculus, and bilateral arcuate fasciculi
Sepulcre et al., 2009 [29]	54 MS	T2 (VLSM)	Correlation with bilateral parieto-frontal, centrum semiovale, temporo-occipital white matter, internal capsule, right pontomesencephalic tegmentum, right cerebellar peduncle, and right anterior cingulate
Van Hecke et al., 2010 [26]	20 MS	DTI	Correlation with fractional anisotropy in left inferior longitudinal fasciculus, forceps minor, internal and external capsule, corpus callosum, left cingulum, superior longitudinal fasciculus, and corona radiate
Nocentini et al., 2012 [9]	18 MS	T1	No significant correlations
Yu et al., 2012 [27]	37 RRMS	DTI	Correlation with reduced fractional anisotropy in sagittal striatum, posterior thalamic radiation, and external capsule
Sbardella et al., 2013 [8]	36 RRMS	T1 and DTI	Correlation with orbitofrontal cortex, and white matter tracts including the corpus callosum, internal capsule, posterior thalamic radiations, and cerebral peduncles
D’haeseleer et al., 2013 [30]	18 MS	Arterial spin labelling	Correlation between PASAT performance and cerebral blood flow in the left centrum semiovale
Baltruschat et al., 2015 [31]	17 RRMS	T1 and fMRI	No significant correlation between PASAT performance and functional connectivity in the MS group
Riccitelli et al., 2017 [10]	177 RRMS	T1 (VBM) and DTI (TBSS)	Correlation with atrophy of the bilateral thalamus, caudate and putamen, right anterior cingulate, right superior frontal gyrus, and right precentral, left superior temporal, and right fusiform gyri. Correlation with reduced fractional anisotropy and increased mean diffusivity in several white matter tracts
Present study	242 MS	T1 (VBM) and FLAIR (VLSM)	Correlation with bilateral precuneus and posterior cingulate, bilateral caudate and putamen, and bilateral anterior cerebellum

*RRMS* relapsing-remitting multiple sclerosis, *MS* multiple sclerosis, *DTI* diffusion tensor imaging, *TBSS* tract-based spatial statistics, *VLSM* voxel-based lesion symptom mapping; *fMRI* functional magnetic resonance imaging, *VBM* voxel-based morphometry, *FLAIR* fluid-attenuated inversion recovery

In addition, we also observed a significant correlation between PASAT performance and 3 subcortical regions: caudate, putamen, and cerebellum. This emphasises the role of the basal ganglia and cerebellum in cognitive disorders in MS [27], and specifically in PASAT performance. The role of subcortical structures in cognitive disorders is increasingly recognised, with several structures participating in cognitive and behavioural functions through their connections with the cortex [28].

Lesion symptom mapping found several regions associated with poorer PASAT performance. In this regard, white matter lesions in the left cingulum, corpus callosum, corticospinal tract, and arcuate fasciculus were associated with poorer performance. These findings are similar to those of previous studies using diffusion tensor imaging (DTI), where multiple white matter tracts were associated with PASAT performance [8, 10, 29, 30]. Interestingly, whole-brain voxel-based analysis did not show any significant results, and ROI-based analyses lost statistical significance when total white matter lesion volume was included as a covariate in the statistical model. This may suggest that PASAT performance is influenced to a greater extent by the total lesion volume than by specific lesions in particular white matter regions and tracts. Analogously, previous studies using DTI have also found white matter impairment to have a secondary role in PASAT performance, in comparison to grey matter atrophy [10, 31].

Regarding whole-brain measures, our study found significant associations between PASAT performance and total white matter lesion volume, and raw grey and white matter volumes, but not normalised brain volumes. Although some correlations were statistically significant, the size of the correlation was small. This suggests a minor influence of these MRI measures in cognitive test performance, and statistical significance may be probably explained because of the large sample size included in this study. Previous studies have found conflicting results; a meta-analysis conducted in 2014 could not establish a definitive conclusion regarding the correlation between whole MRI findings and PASAT performance due to missing data and the heterogeneity of the studies [6]. Therefore, our findings, with a weak or non-significant correlation, support the search of brain regions as a better approach to explaining the pathophysiology of impaired PASAT performance and, thus, of impairment of the cognitive functions involved in the performance of this test in MS. However, the correlation between PASAT and global brain volumes could also be interpreted as a role of brain reserve in maintaining PASAT performance, as has been suggested previously [32].

In our study, PASAT results showed impairment in 31% of patients, a similar percentage to that found in previous studies [2, 10]. PASAT performance was correlated with most of tests of the neuropsychological battery examining several cognitive domains. This confirms the usefulness of PASAT as a general test in MS that may be applied as a neuropsychological screening test. However, the size of the correlation with most tests of memory, language, visuospatial functioning etc. was generally low. Conversely, PASAT was moderately correlated with several time-dependent neuropsychological tests, especially those associated to attention and executive functioning. Regarding fatigue and depression, the correlation with PASAT was low. This weak correlation suggests that fatigue and depression has a little influence in PASAT performance and, thus, impairment in this test is more related to cognitive issues than non-cognitive factors. Indeed, VBM analysis controlling for depression displayed the same brain regions associated to the PASAT performance.

PASAT involves several cognitive functions, including auditory perception and processing, speech production, mathematical abilities, working memory, several components of attention and concentration, processing capacity, and information processing speed [5, 33]. This suggests that PASAT, like almost all neuropsychological tests, should not be considered a measure of a single cognitive function (i.e. information processing speed) [5]. In the specific setting of MS, our results suggest that PASAT performance is associated with the status of several brain regions (posterior cingulate/precuneus, basal ganglia, and cerebellum), probably involved in the fronto-subcortical and default mode networks. White matter lesions may contribute to pathophysiology, but we could not find specific localisations associated with performance in the test. Overall, our findings support the status of PASAT as a test associated with information processing speed, among others cognitive functions. However, because correlation with other time-dependent neuropsychological tasks was moderate, information processing speed should not be regarded as a unitary concept. From this perspective, PASAT may be a measure of the efficiency of cognitive effort and concentration during a high-demand attentional task requiring the preservation of both cortical and subcortical structures; information processing speed may represent the level of efficiency that the patient achieves.

Our study has some limitations. Firstly, we included only 3D T1-weighted and FLAIR sequences, but not such other sequences of interest as DTI or fMRI. Thus, hypotheses about the brain networks involved in the execution of the test are speculative. Although we use findings from previous studies using these techniques, a multimodal MRI study of the same sample would be highly informative. Secondly, we included only patients who completed the PASAT, which may represent a selection bias. However, due to the large sample size and the clinical and demographic characteristics of the sample, we believe that our cohort of patients is representative of MS. Another interesting future point would be to examine the neural correlates of PASAT performance in each form of MS, in order to search potential differences between relapsing remitting and progressive variants [34]. Finally, our study has a cross-sectional design. Longitudinal studies may be of interest to better understand the dynamics of cognitive dysfunction in patients with MS.

## Conclusions

Our study suggests that, on the one hand, the neural basis of PASAT performance involves the posterior cingulate/precuneus, probably associated with default mode network and participating in attention. On the other hand, the test is also correlated with several subcortical structures (particularly caudate, putamen, and cerebellum), which probably contribute to automation and behavioural adjustments during test performance. This emphasises the role of both cortical and subcortical structures in cognitive functioning and information processing speed in MS.

## Additional file

Additional File 1: **Table S1.** Voxel-Based Morphometry Analysis. Multiple regression analysis showing correlations between PASAT and brain regions, using age, sex, years of education, MRI sequence, total intracranial volumen and depression as covariates. FDR corrected *p*-value < 0.05, k = 100. (DOCX 82 kb)

## Abbreviations

BNT: Boston naming test
BRN-B: Brief repeatable neuropsychological battery
DTI: Diffusion tensor imaging
EDSS: Expanded disability status scale
FCSRT: Free and cued selective reminding test
fMRI: functional magnetic resonance imaging
JLO: Judgement of line orientation
MRI: Magnetic resonance imaging
MS: Multiple sclerosis
PASAT: Paced auditory serial addition test
ROCF: Rey-Osterrieth Complex Figure
ROI: Region of interest
SD: Standard deviation
SPM: Statistical parametric mapping
TMT: Trail making test
ToL: Tower of London-Drexel version
