# Supplementary material for: Structural MRI correlates of PASAT performance in multiple sclerosis
Source: BMC Neurol. 2018 Dec 20;18:214. doi: 10.1186/s12883-018-1223-0 (PMC6300910; doi:10.1186/s12883-018-1223-0)
Supplement: Supplementary file 1 — Table S1. Voxel-Based Morphometry Analysis. Multiple regression analysis showing correlations between PASAT and brain regions, using age, sex, years of education, MRI sequence, total intracranial volumen and depression as covariates. FDR corrected p-value < 0.05, k = 100. (DOCX 82 kb) [file 12883_2018_1223_MOESM1_ESM.docx]

| **Table. Voxel-based morphometry analysis**. Multiple regression analysis showing correlations between PASAT and brain regions, using age, sex, years of education, MRI sequence, total intracranial volumen and depression as covariates.  FDR corrected *p*-value <0.05, k=100. | | | | | | | | |
| --- | --- | --- | --- | --- | --- | --- | --- | --- |
| *Brain region (Brodmann area)* | *MNI coordinates* | | | *T value* | *Z score* | *Cluster-level* | *Peak-level* | *K (number of voxels)* |
|  | *x* | *y* | *z* |  |  | p-value (FWE corrected) | p-value (FDR-corrected) |  |
| Left and right precuneus and posterior cingulate (7, 31) | -4 | -48 | 45 | 5.31 | 5.15 | <0.0001 | 0.009 | 3156 |
|  | 4 | -31 | 42 | 4.83 | 4.71 |  | 0.009 |  |
|  | 4 | -63 | 18 | 4.27 | 4.19 |  | 0.013 |  |
| Right cerebellum | 24 | -46 | -14 | 5.02 | 4.89 | <0.0001 | 0.009 | 2268 |
|  | 24 | -57 | -12 | 5.02 | 4.88 |  | 0.009 |  |
| Right caudate and insula | 34 | 2 | -3 | 4.94 | 4.81 | <0.001 | 0.009 | 1751 |
| Left cerebellum | -27 | -49 | -24 | 4.40 | 4.31 | 0.026 | 0.011 | 868 |
| Left precuneus (7) | -6 | -70 | 45 | 4.30 | 4.21 | 0.432 | 0.013 | 244 |
|  | -9 | -64 | 34 | 3.76 | 3.70 |  | 0.031 |  |
| Left caudate and insula | -32 | 3 | -3 | 4.19 | 4.11 | 0.040 | 0.015 | 768 |
|  | -24 | 18 | 4 | 3.98 | 3.91 |  | 0.021 |  |
|  | -40 | -10 | 1 | 3.89 | 3.82 |  | 0.025 |  |
